# Drug tapering in animal research: current practices and challenges

**DOI:** 10.3389/fphar.2025.1544784

**Published:** 2025-08-13

**Authors:** Yatong Zhang, Mengrui Chen, Tongtong Jiang, Qianfang Fu, Rong Ma, Lianghui Nie, Yuqiong You, Shuqi Gao, Ping Rong

**Affiliations:** ^1^ Department of Pediatrics, First Teaching Hospital of Tianjin University of Traditional Chinese Medicine, Tianjin, China; ^2^ Department of Pediatrics, National Clinical Research Center for Chinese Medicine Acupuncture and Moxibustion, Tianjin, China

**Keywords:** animal experiments, drug tapering, tapering timing, tapering methods, tapering outcomes

## Abstract

**Purpose:**

To synthesize and summarize the scope and protocols of drug tapering in existing animal studies, providing a reference for standardized tapering interventions in future research.

**Methods:**

Utilizing a scoping review framework, a systematic search was conducted in PubMed, Web of Science, China National Knowledge Infrastructure (CNKI), Wanfang Data Knowledge Service Platform (Wanfang), China Science and Technology Journal Database (VIP), and China Biomedical Literature Service System (SinoMed) for studies on drug tapering in animal experiments published up to May 1, 2024. Key data such as disease type, drugs used, and tapering methods were extracted and analyzed.

**Results:**

Nineteen studies were included, addressing a range of diseases including respiratory issues, oncology, nephrology, neuropsychiatric disorders, fractures, organ transplantation, and perioperative pain. Only one study commenced tapering upon the emergence of drug toxicity, while the others did not explicitly state the timing of tapering. Tapering methods identified included reducing single drug doses, decreasing administration frequency, and intermittent dosing. The outcomes of tapering were classified into drug discontinuation, low-dose maintenance, and drug substitution, with all studies reporting the effects post-tapering.

**Conclusion:**

The methods of drug tapering in animal studies are influenced by multiple factors, including disease characteristics, treatment duration, animal tolerance, and specific goals. There remains a need for further refinement in tapering schedules, methods, and milestones, and the establishment of relevant evaluation metrics to better define tapering timing.

## 1 Introduction

Drug tapering involves the gradual reduction of the total daily dose or the extension of the dosing interval until the drug is discontinued during disease treatment. This process consists of two stages: drug reduction and drug withdrawal. The primary goals of drug tapering are to mitigate adverse effects, minimize the risk of disease recurrence, and maintain therapeutic efficacy. In April 2024, a study published in the Annals of the Rheumatic Diseases, focused on drug reduction in stable IgG4-related disease (IgG4-RD) (Peng et al., 2024). The study highlighted the importance of maintenance therapy in preventing disease relapse. While there is a consensus on the clinical management of many diseases, the standardization of drug reduction and withdrawal remains a challenging unresolved issue. Therefore, it is crucial to develop a meticulous and rational strategy for reducing and withdrawing drugs that require prolonged usage or exhibit significant toxicities. Such an approach aims to ensure safe tapering, reduce relapse rates, improve compliance, and alleviate financial burdens, ultimately benefiting patients.

Animal studies, as the cornerstone of preclinical research, serve as a critical bridge between basic science and clinical applications. Data from these studies provide a foundation for evidence-based decision-making (Xiong et al., 2005), underscoring the necessity of preclinical drug tapering investigations. Several factors contribute to the low translation rate from basic science to clinical practice, including internal validity (scientific study design, execution, analysis, and reporting) and external validity (the generalizability of results from one setting or species to other contexts and populations) (Pound and Ritskes-Hoitinga, 2018; Bailoo et al., 2014). Improving internal validity has been shown to enhance the rate of clinical translation (Dirnagl and Endres, 2014). By constructing animal models that closely mimic human disease characteristics, implementing drug tapering interventions similar to clinical settings, and developing comprehensive tapering strategies tailored to experimental needs or the animals’ responses to treatment, researchers can create more scientifically rigorous and standardized studies. This approach enhances the potential clinical translational value of the results.

Additionally, conducting clinically appropriate tapering studies in animal research can maximize animal welfare. In 2010, the ARRIVE guidelines (Kilkenny et al., 2010) for improving the reporting of animal experiments emphasized the importance of generalizability *in vivo* experiments, particularly their relevance to human biology and diseases. The “Guide for the Care and Use of Laboratory Animal” (National Research Council of the National Academies, 2011), published in the United States in 2012, also emphasized that the management of laboratory animals includes their production, use, protection, care, and ethical considerations. The currently accepted ethical standard for regulating and reviewing animal experimentation is the 3Rs principle (Replacement, Reduction, Refinement) (Russell and Burch, 1959). Tapering studies are often based on clinical experience or the animal’s response to the drug, following a gradual and slow approach. During the experiment, it is essential to closely observe changes in the animal’s behavior, conduct indicator tests, and take timely appropriate actions, aligning with the principles of the 3Rs or the 4Rs (Ogden, 1996).

Despite the importance of conducting tapering studies in animals, the current number of such studies is limited and heterogeneous. To encompass a broader range of evidence types, this paper adopts a scoping review approach to describe the scope and methods of tapering in existing studies. Additionally, it proposes further research strategies to inform the design of future tapering experiments and facilitate the translation of clinical needs.

## 2 Materials and methods

The review is conducted according to the PRISMA-ScR guidelines (Tricco et al., 2018), utilizing the methodological framework for scoping reviews proposed by Arksey and O’Malley (2005) and updated by JBI (Peters et al., 2021).

### 2.1 Search strategy

A systematic search of PubMed, Web of Science, CNKI, Wanfang, VIP, and SinoMed was conducted from database inception to May 1, 2024. For the English databases, a combination of subject and free-text search methods was used, while for the Chinese databases, a combination of subject and abstract searches was employed. The search strategy incorporated terms related to rats, mice, animal experiments, and drug tapering. The complete search strategy can be found in Supplementary Material S1. As rodents are the most widely used species in animal testing, this study focuses on rodent testing.

### 2.2 Inclusion criteria

1. The study type was animal research with clear outcome measures.
2. The intervention included the process of drug tapering.
3. The drugs used were either marketed varieties or herbal medicines.
4. The language was either Chinese or English.

### 2.3 Exclusion criteria

1. Interventions involving immediate drug withdrawal without a tapering process.
2. Drug tapering is only reflected in the modeling.
3. Veterinary and agricultural studies.
4. Literature inconsistent with the study type, such as cellular experiments, clinical trials, theoretical discussions, and reviews.
5. Repeatedly published studies, retaining only the one with the most comprehensive information.

### 2.4 Study selection

The search results were imported into EndNote X9.1 to eliminate duplicates. Two researchers independently read the titles, abstracts, and full texts in turn for screening, cross-comparing results, and consulting a third investigator to resolve any disagreements.

### 2.5 Data extraction and quality control

One researcher performed data extraction by importing literature that met the criteria into Microsoft Excel 2019, and another reviewer verified the extraction. The data included information such as the year of publication, title, authors, disease, animal, drug, tapering timing, tapering methods, tapering cycle, and tapering outcomes. Disagreements were resolved through discussion between the two reviewers or by consulting a third investigator.

### 2.6 Results synthesis and presentation

The results were summarized using descriptive statistics and presented in both tabular and narrative forms, outlining the range of applications and specific tapering strategies.

## 3 Results

### 3.1 Study screening

**FIGURE 1:**
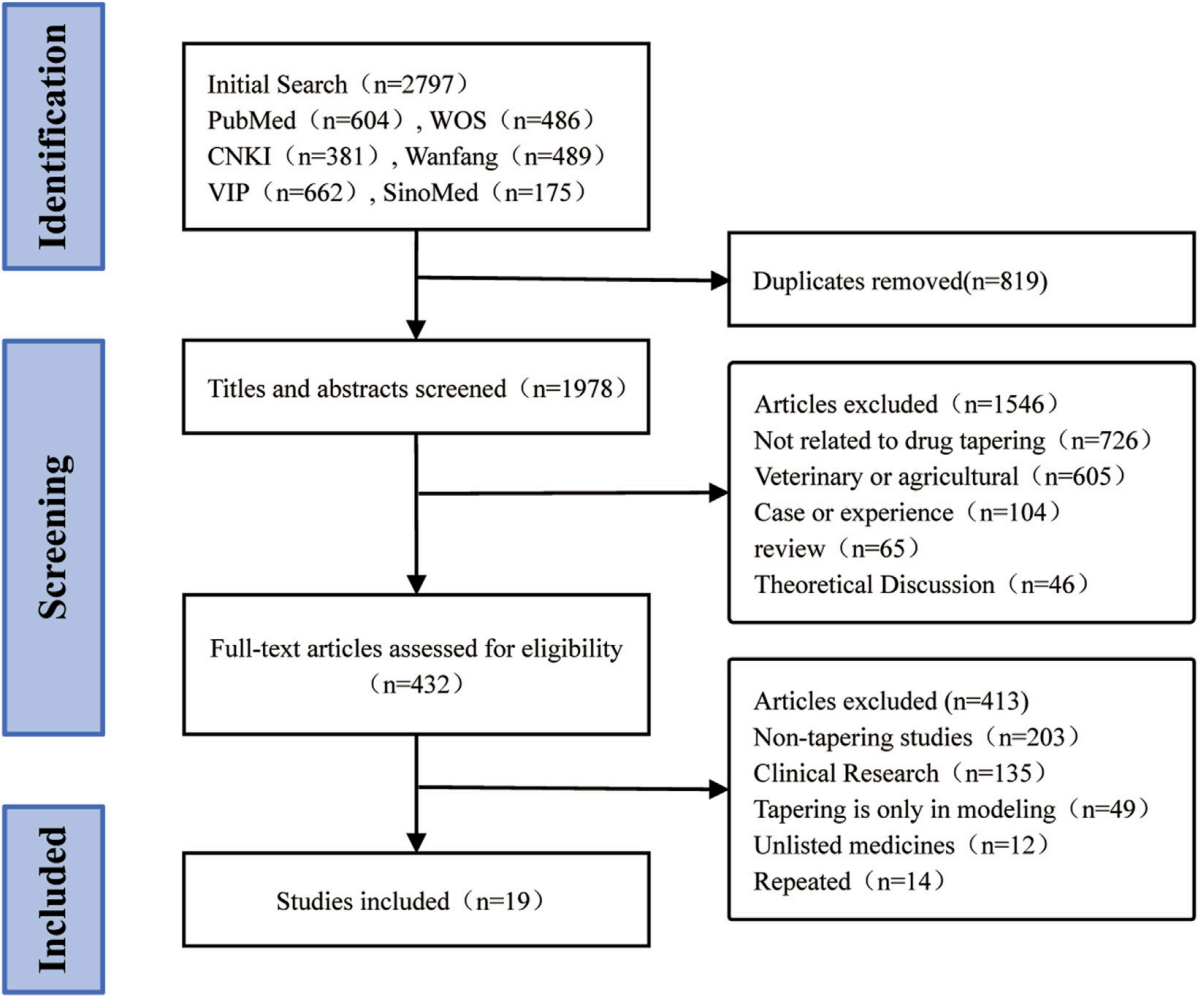
Flowchart of the literature screening process.

A total of 2,797 articles were retrieved. After excluding 819 duplicate articles and screening titles and abstracts, 1,546 articles were further excluded. Among the 432 full-text articles screened, 203 were non-tapering studies, 135 were clinical tapering studies, 49 only described tapering methods in modeling, 12 involved non-marketed drugs, and 14 were duplicate articles. Ultimately, 19 animal studies were included. The literature screening process is shown in Figure 1.

### 3.2 Study characteristics

The experimental animals used in this review included Wistar rats, SD rats, DA rats, Lewis rats, C57BL/6 mice, BALB/C mice, and nude mice. Study types included pharmacodynamic and toxicity studies in diseases such as respiratory conditions, cancer, renal issues, fractures, liver transplantation, perioperative pain, and neuropsychiatric disorders. A wide range of drug tapering categories was examined, including hormones, immunosuppressants, targeted agents, glucose- and lipid-lowering drugs, antiepileptics, and opioids. The characteristics of the included studies are shown in Table 1.

**TABLE 1:** Study characteristics.

Study	Animal	Disease	Drug	Timing	Methods	Cycle	Outcome
Su et al. (2014)	BALB/C mice	Asthma	Prednisone, Wuzi Sanhuang Formula	42nd day	Prednisone reduced by 0.325 mg/kg every 2 days	3 weeks	①
Liu et al. (2014)	SD rats	Asthma	Dexamethasone, Epimedium and Ligustrum	50th day	Dexamethasone reduced by 0.1 mg/kg per week	4 weeks	①
He (2006)	SD rats	Asthma	Dexamethasone, Wumei pills	3rd week	Dexamethasone reduced by 0.1 mg/kg per week	4 weeks	①
Yan (2016)	SD rats	Asthma	Dexamethasone, Sanbu Formula	3rd week	Dexamethasone reduced by 0.1 mg/kg per week	4 weeks	①
Wang (2010)	SD rats	Asthma	Dexamethasone, Yougui Pills	16th day	Dexamethasone dose halved	2 weeks	②
Zhou et al. (2019)	SD rats	Fracture	Simvastatin	8th day	Decrease by 2.5 mg/kg per week	4 weeks	②
Li (2018)	SD rats	Fracture	Simvastatin	8th day	Decrease by 2.5 mg/kg per week	4 weeks	②
Grace et al. (2019)	SD rats	Perioperative pain	Morphine	8th day	Daily dose halved	3 days	②
Zhu et al. (2010)	DA/Lewis rats	Liver transplantation	Tacrolimus	8th day	Daily dose halved	5 days	①
Kaur et al. (2013)	SD rats	Diabetes	Glipizide, Annona squamosa	11th day	Glipizide reduced by 1.25 mg/kg every 10 days	30 days	②
Hodsman et al. (1999)	SD rats	Osteoporosis	Raloxifene analogue、hPTH(1–34)	After 12 weeks	hPTH (1–34) 5 times per week reduced to 2 times per week	8 weeks	②
Bozec et al. (2010)	nude mice	Tumor	Cetuximab, Gefitinib	7 days later	Both reduced by half daily	7 days	②
Macenski et al. (1994)	Wistar rats	Substance use disorders	Methadone, Buprenorphine	31st day	Methadone 31-day reduction of 1.5 mg/kg	74 days	①
Yang et al. (2023)	Wistar rats	Epilepsy	Carbamazepine, Banxia Baizhu Tianma Decoction	7th week	Carbamazepine discontinued, herbal medicine maintenance	6 weeks	①
Chen et al. (2010)	SD rats	Nephrotic syndrome	Prednisone, Shenkang-Ⅱ	3rd week	Prednisone reduced by 0.5 mg/kg per day	2 weeks	②
Julian and Iwamoto (2021)	SD rats	Toxicity studies	Erlotinib	1 week later	70 mg/kg at week 1, discontinued at week 2, reduced to 35 mg/kg at week 3	2 weeks	②
Sereno et al. (2015)	Wistar rats	Toxicity studies	CsA	4th week	30 mg/kg for the first 3 weeks, reduced to 5 mg/kg for the second 3 weeks, and to 1 mg/kg of sirolimus for the last 3 weeks	6 weeks	③
Sbiera et al. (2011)	C57Bl6 mice	Toxicity studies	Dexamethasone	4 days later	10 mg/L on days 4–7, 5 mg/L on days 8–11 and 1 mg/L on days 12–14	7 days	②
Akar (2005)	Wistar rats	Toxicity studies	Tacrolimus	toxic effects occur	Amount halved	2 months	②

Note: ①, Discontinuation; ②, Low dose maintenance; ③, Medication substitution.

### 3.3 Timing of drug tapering

Due to the significant heterogeneity among different diseases, the timing of drug tapering cannot be universally prescribed. Reducing medication too early may diminish the treatment’s effectiveness and increase the risk of recurrence, while delaying the reduction may lead to various adverse reactions and heighten the economic burden. The appropriate timing for medication reduction occurs after the condition has stabilized and symptoms have been alleviated (Peng et al., 2024); however, existing animal studies have not described specific evaluation methods for this process.

In five asthma studies, glucocorticoid tapering began at week three in three studies (He, 2006; Yan, 2016; Wang, 2010), and on day 42 and day 50 in two studies (Su et al., 2014; Liu et al., 2012). This timing generally falls in the middle or late stages of the total treatment course. For surgical diseases such as fractures, perioperative pain, and liver transplantation, drug tapering occurred on the 8th day (Zhou et al., 2019; Li et al., 2019; Grace et al., 2019; Zhu et al., 2010), without a clear pattern. In models of diabetes (Kaur et al., 2013) and substance use disorder (Macenski et al., 1994), dosage reduction commenced at the beginning of administration. For osteoporosis models (Hodsman et al., 1999), dosage reduction occurred in the middle and late stages of treatment. Experimental studies on epilepsy (Yang et al., 2023), tumors (Bozec et al., 2010), and nephrotic syndrome (Chen et al.) all initiated dosage reduction in the middle stage of administration. Toxicity studies involving erlotinib, cyclosporine, and dexamethasone began reduction in the early stage of administration (Julian and Iwamoto, 2021; Sereno et al., 2015; Sbiera et al., 2011). One study detailed the specific timing of dose tapering in a tacrolimus toxicity study, where the dosage was reduced upon the occurrence of toxic side effects (Akar et al., 2005).

### 3.4 Tapering method

Drug tapering methods include reducing the single drug dose, reducing the number of administrations, and intermittent administration. Reducing the single drug dose can be further categorized into fixed-dose reduction, halving the dose, or irregular tapering. Among the 19 included studies, 8 (42.1%) used a regularly reduced fixed dose every week/day (Su et al., 2014; Liu et al., 2012; He, 2006; Yan, 2016; Zhou et al., 2019; Li et al., 2019; Kaur et al., 2013; Chen et al., 2010), 5 (26.3%) studies sequentially halved the dose (Wang, 2010; Grace et al., 2019; Zhu et al., 2010; Bozec et al., 2010; Akar et al., 2005), 4 (21.1%) studies followed the principle of reducing the dose quickly at first and then slowly (Macenski et al., 1994; Yang et al., 2023; Sereno et al., 2015; Sbiera et al., 2011), 1 (5.3%) study reduced the number of dosing times (Hodsman et al., 1999), and 1 (5.3%) study administered the drug intermittently (Julian and Iwamoto, 2021). In this study, tapering of glucocorticoids, simvastatin, and glipizide followed regular fixed dose reductions. Morphine, tacrolimus, cetuximab, and gefitinib doses were halved. hPTH (1–34) was reduced from 5 times a week to 2 times a week, achieved by reducing the number of dosing times. In the toxicity study of erlotinib, intermittent administration—one week on, 1 week off, and 1 week of dose reduction—was used to observe the occurrence of adverse reactions.

In efficacy studies involving animal experiments, it is observed that glucocorticoids should be gradually reduced over weeks or days. The speed of safe reduction depends on the stability of the disease and the risk of inhibiting the HPA axis. There is no fixed rule for tapering immunosuppressants and targeted drugs, but they generally reduce quickly at first and then slowly. The tapering design in toxicity studies varies more, including intermittent dosing, replacement dosing, and half-dose reductions, all formulated based on target needs and the tolerance of experimental animals.

### 3.5 Tapering outcome

The outcomes of drug tapering include drug withdrawal, low-dose maintenance, and drug replacement. In this study, 7 (36.8%) experiments achieved complete drug withdrawal (Su et al., 2014; Liu et al., 2012; He, 2006; Yan, 2016; Zhu et al., 2010; Macenski et al., 1994; Yang et al., 2023), 11 (57.9%) experiments reduced to a lower dose for maintenance (Wang, 2010; Zhou et al., 2019; Li et al., 2019; Grace et al., 2019; Kaur et al., 2013; Hodsman et al., 1999; Bozec et al., 2010; Chen et al., 2021; Julian and Iwamoto, 2021; Sbiera et al., 2011; Akar et al., 2005), and 1 (5.3%) experiment completed drug replacement (Sereno et al., 2015). Relapse and withdrawal reactions after drug tapering are the focus of clinical attention and are important indicators of the success of drug tapering (Jingyou and Li, 2023; Meng et al., 2023; Zhou and Lin, 2022). Generally, reducing medication from a sufficient amount to low-dose maintenance may benefit patients more. Complete discontinuation of medication carries the risk of reduced efficacy or recurrence. If the goal is rapid medication reduction, drug replacement or adding traditional Chinese medicine treatment should be considered. Drug replacement involves substituting drugs with greater toxic side effects for those with fewer side effects while maintaining efficacy, such as replacing cyclosporine with sirolimus (Sereno et al., 2015). Adding traditional Chinese medicine is an effective method for achieving rapid withdrawal of target drugs. Among the 7 experiments on combined traditional Chinese and Western medicine treatment in this study, 6 maintained traditional Chinese medicine treatment after reducing and stopping the target drugs, indicating that traditional Chinese medicine treatment can help achieve rapid withdrawal of chemical drugs. However, the studies did not describe the maintenance time of traditional Chinese medicine or replacement drugs and how to withdraw them.

### 3.6 Efficacy after dose tapering

All studies described the efficacy after drug tapering. In animal experiments involving the combined intervention of Chinese and Western medicine, 6 (31.6%) studies reported that traditional Chinese medicine could maintain therapeutic effects and reduce adverse reactions caused by chemical drugs after gradually stopping Western medicine (Su et al., 2014; Liu et al., 2012; He, 2006; Yan, 2016; Wang, 2010; Chen et al., 2021). The study by Chen et al. (2010) highlighted that the advantages of large-dose and long-term application of traditional Chinese medicine are more evident. Yang et al. (2023) found that using traditional Chinese medicine could effectively facilitate the withdrawal of anti-epileptic drugs. In the treatment of fractures and tumors, a gradual reduction in medication is more beneficial to the disease than maintaining a constant dose (Zhou et al., 2019; Li et al., 2019; Bozec et al., 2010). However, for perioperative pain, drug tapering has not shown any advantage and has failed to shorten the duration of pain (Grace et al., 2019). In the liver transplant model, the medication regimen of clinical patients is simulated at the animal level; the dosage is gradually reduced, changes in blood drug concentration are evaluated, and a more reasonable immunotherapy plan can be explored based on the timing of immune rejection reactions (Zhu et al., 2010). In toxicity studies, the relationship between dose and toxicity can be explored through drug tapering. For example, reducing the dose of erlotinib can prevent the occurrence of rashes (Julian and Iwamoto, 2021). The toxicity of tacrolimus and cyclosporine is dose-dependent (Sereno et al., 2015; Akar et al., 2005), and short-term glucocorticoid application (2 weeks) does not affect normal mouse T cells (Sbiera et al., 2011).

## 4 Discussion

### 4.1 Necessity of conducting drug tapering studies in animal experiments

By gradually tapering specific medications, it is possible to evaluate several aspects: - The advantages and disadvantages of tapering regimens compared to continuous administration. - The differences in efficacy between gradual tapering and abrupt discontinuation. - The adverse reactions to chemical drugs improved through the addition of traditional Chinese medicine. - Optimal strategies for medication tapering. Compared to continuous administration and sudden drug withdrawal, reasonable drug tapering has significant advantages in reducing drug resistance, alleviating side effects, and improving patients’ quality of life. For example, intermittent drug administration in tumors can delay the onset of drug resistance and improve efficacy while reducing adverse reactions (Lin et al., 2022). The tapering of antipsychotic drugs benefits patients with schizophrenia in the long term (Liu et al., 2023). However, the unreasonable withdrawal of drugs for Alzheimer’s disease and epilepsy can exacerbate the progression of these conditions (Gu et al., 2023; Feng, 2013; Zhang, 2015).

### 4.2 Application scope of drug tapering research

For drugs that need to be taken long-term or have significant toxic side effects, a detailed and reasonable tapering strategy should be formulated to determine the appropriate timing, speed, and method of tapering. Drug tapering has a wide range of applications. Based on the results of this review and clinical practice, it can be broadly applied to hormonal drugs, immunosuppressants, targeted therapies, opioid analgesics, neurological drugs, and antiepileptic drugs.

#### 4.2.1 Hormonal drugs

Hormonal drugs consist of medications using human or animal hormones, including organic substances with similar structures and mechanisms, as active ingredients. Glucocorticoids (GC) are the most widely used and serve as first- or second-line treatments for various conditions such as infections (Chinese Education Associa tion of Chronic Airway Diseases China Asthma AllianceChina Asthma Alliance, 2024), allergies (Cardona et al., 2020), immune disorders (Fanouriakis et al., 2024), and kidney diseases (Kidney Disease: Improving Global Outcomes KDIGO Glomerular Diseases Work Group, 2021). Expert consensus indicates that for GC regimens exceeding 7 days, the medication should be tapered before discontinuation, following a “fast first, then slow” principle (Consensus Expert Group on the Emergency Use of Glucocorticoids, 2020). Given the adverse events associated with long-term GC use—such as infections, ulcers, fractures, and severe impacts on growth and development in pediatric patients—expedited tapering and discontinuation are necessary. The current consensus emphasizes prompt reduction and cessation of GC, as in rheumatoid arthritis, where short-term use is advised, and tapering off should occur when clinically feasible (Smolen et al., 2023). In systemic lupus erythematosus, reducing to a maintenance dose of ≤5 mg/day after stabilization, with complete discontinuation as the goal, is recommended (Fanouriakis et al., 2024). However, there remains a lack of specific tapering and discontinuation guidelines, highlighting the need for further research.

#### 4.2.2 Immunosuppressive agents

Immunosuppressants inhibit the proliferation and function of immune-related cells, such as T cells and B cells, reducing the antibody immune response. They are primarily used to prevent rejection in organ transplantation and treat autoimmune diseases (Gao and Liu, 2023; Chinese Society of Organ Transplantation, 2019). Organ transplantation is an effective method to improve end-stage organ failure. To reduce post-transplant rejection, immunosuppressants are typically administered in the pre-transplant or early post-transplant period (Taylor et al., 2005). However, in transplant recipients, a strong immunosuppressive state can disrupt the immune surveillance system and promote disease recurrence, while insufficient immunosuppression may lead to rejection. There is no unified strategy or monitoring method to maintain this balance. Currently, complete withdrawal of immunosuppressants is not recommended for transplant recipients; instead, individualized low-dose immunosuppressive regimens are advocated (Chen et al., 2021; Shi and Yuan, 2016).

Additionally, immunosuppressants are widely used in treating various autoimmune diseases. However, due to their lack of selectivity and specificity, they suppress both normal and abnormal immune functions, leading to a range of infectious or non-infectious adverse events and significant hepatotoxicity and nephrotoxicity (Mo and Zheng, 2024-10). Therefore, further research is needed to explore the relationship between dosage and toxicity through tapering strategies and to find a balance between therapeutic efficacy and toxicity.

#### 4.2.3 Targeted drugs

Targeted drugs, emerging from the precision medicine era, are widely accepted for treating cancer patients due to their high efficacy and low toxicity (Bedard et al., 2020). However, they can cause multi-system toxic side effects, often appearing early in treatment (Kalemkerian et al., 2018). For instance, bevacizumab may lead to endothelial dysfunction and arterial adverse events, requiring early blood pressure monitoring (Totzeck et al., 2017). Apatinib can raise blood pressure, increase bleeding risk, and cause proteinuria, bone marrow suppression, and liver damage (Zhu et al., 2021). The severity of these side effects generally increases with cumulative doses, limiting clinical use. Therefore, the relationship between dosage and toxicity needs further evaluation, and dose reduction strategies should be employed when feasible to reduce adverse events and enhance tolerance.

Some patients develop resistance during treatment (Ottaviano et al., 2021; Ladd et al., 2024), but changing the administration method can mitigate this. Re-administering the drug after a prolonged withdrawal can restore tumor sensitivity (Das Thakur et al., 2013). Studies indicate that intermittent dosing can delay melanoma resistance, achieving better outcomes (Kong et al., 2017), while tapered dosing is more effective than constant dosing in inhibiting head and neck tumor growth (Bozec et al., 2010). Targeted drugs will continue to play a key role in cancer and autoimmune disease treatment. Further exploration of rational and effective dose reduction strategies is needed.

#### 4.2.4 Opioid analgesics

Opioid analgesics are recognized as the most effective treatment for moderate to severe pain and are recommended by the World Health Organization for cancer pain management and acute pain control. Opioid-based regimens have become a global standard for both non-cancer and chronic pain. However, their non-analgesic effects, especially respiratory depression and potential dependence, remain significant challenges. The incidence of opioid-induced respiratory depression is about 1.5% in hospitals and can reach 25% in opioid-addicted patients (Cai et al., 2024). During perioperative care, extensive opioid use can negatively impact long-term outcomes, such as increasing readmission rates by 30% (Long et al., 2018), and may delay recovery due to adverse reactions (Qian, 2024).

Recently, there has been focus on gradual postoperative opioid tapering regimens, although approaches vary. Ma et al. (2024) showed that sequential remifentanil withdrawal can alleviate early postoperative pain in cervical surgery and reduce sufentanil use without affecting recovery quality. Zhang et al. (2022) found that stepwise remifentanil withdrawal can elevate pain thresholds and reduce nausea, vomiting, and agitation. However, Grace, P. M.'s research suggests that tapering does not prevent prolonged postoperative pain (Grace et al., 2019). The optimal tapering methods and schedules still require foundational experimental data to develop the best clinical strategies and avoid misuse.

#### 4.2.5 Psychotropic drugs

The “Pharmacopoeia of the People’s Republic of China: Clinical Medication Guidelines for Chemical Drugs and Biological Products (2010 Edition)” classifies psychoactive drugs into five categories: antipsychotic drugs, antidepressants, anxiolytics, mood stabilizers, and psychostimulants, including 60 varieties such as chlorpromazine, amitriptyline, alprazolam, and atomoxetine. Psychoactive drugs often require long-term treatment, and when the body adapts to the drug, withdrawal reactions can occur if the drug is eliminated faster than the adaptation fades (Reidenberg, 2011). Irrational discontinuation of any psychoactive drug can lead to withdrawal reactions, causing physical and psychological discomfort and potentially triggering disease relapse (Baldessarini and Tondo, 2019).

Horowitz and Taylor (2022). mapped the relationship between different tapering approaches and the risk of withdrawal reactions. Psychoactive drugs should be tapered slowly over a sufficient period to allow the body’s adaptation to dissipate, minimizing withdrawal and relapse risks. Current guidelines encourage reducing antipsychotic drugs to the lowest effective dose but do not specify effective tapering methods (Zhao and Shi, 2015; Li and Ma, 2017). PET imaging shows a hyperbolic relationship between psychoactive drug doses and D2 receptor occupancy, implying that dose reduction should follow a hyperbolic pattern to evenly decrease the dose, aiming for a 5% or 10% decrease in D2 receptor blockade (Horowitz et al., 2021). This tapering method theoretically reduces the risk of withdrawal reactions and relapse after discontinuing antipsychotic drugs. However, this approach requires further evaluation through animal experiments and clinical studies.

#### 4.2.6 Antiepileptic drugs

Despite significant advancements in epilepsy treatment, including neurostimulation, ketogenic diets, surgery, and gene therapy, antiepileptic drugs (AEDs) remain the primary choice for most patients. With AED treatment, 60%–70% of patients achieve seizure control (China Association Against Epilepsy, 2015). It is generally considered feasible to begin tapering medication after more than two seizure-free years; however, improper tapering can lead to recurrence or drug resistance (Lamber et al., 2015).

Our clinical experience supports a slow and gradual reduction in AEDs, but opinions on tapering vary. Strozzi, I (Strozzi et al., 2015) suggests tapering in children after more than two seizure-free years, while Karalok et al. (2020) notes that less than three seizure-free years in children increases relapse risk. For adults, a longer period (over 5 years) is recommended before tapering (Vorderwülbecke et al., 2019). The Italian League Against Epilepsy advises slow tapering (≥6 months) (Beghi et al., 2013), while Ayuga Loro, F. found no difference between rapid (≤3 months) and slow (>3 months) tapering (Ayuga Loro et al., 2020). Duy, P. Q. suggests quickly reducing AEDs to one-third of the dose rather than discontinuing (Duy et al., 2020). In clinical practice, psychiatric comorbidities in children with epilepsy occur nearly five times more frequently than in the general population (Dagar and Falcone, 2020). When AEDs are combined with psychiatric medications, more cautious tapering strategies are needed.

## 5 Limitations and prospects of existing studies

Current animal studies often lack comprehensive evaluation of the appropriate timing for drug tapering and do not provide sufficient data on relapse or subsequent treatment after discontinuation. Additionally, these studies have not investigated the tapering of traditional Chinese medicine in cases where Chinese and Western medicines are used in combination. Future research should emphasize the practicality and completeness of tapering protocols by defining clear timing for drug reduction, establishing relevant evaluation metrics, and setting detailed tapering milestones. After tapering, an adequate observation period should be maintained to assess prognosis and gather data on relapse and treatment resumption.

In the selection of experimental animals, rodents, livestock, and non-human primates can be chosen according to the type of disease being studied. Overall, the design of tapering experiments should comprehensively consider disease characteristics, animal species, treatment duration, and experiment type. It is also recommended that further studies focus on the tapering and withdrawal of traditional Chinese medicine.

## Data Availability

The original contributions presented in the study are included in the article/Supplementary Material, further inquiries can be directed to the corresponding author.

## References

[B1] Akar Y. Yucel G. Durukan A. Yucel I. Arici G. (2005). Systemic toxicity of tacrolimus given by various routes and the response to dose reduction. Clin. Exp. Ophthalmol. 33 (1), 53–59. 10.1111/j.1442-9071.2005.00942.x 15670079

[B2] Arksey H. O’Malley L. (2005). Scoping studies: towards a methodological framework. Int J Soc Res Methodol. Theory & Pract 8 (1), 19–32. 10.1080/1364557032000119616

[B3] Ayuga Loro F. Gisbert Tijeras E. Brigo F. (2020). Rapid versus slow withdrawal of antiepileptic drugs. Cochrane Database Syst. Rev. 1 (1), CD005003. 10.1002/14651858.CD005003.pub3 31990368 PMC6986471

[B4] Bailoo J. D. Reichlin T. S. Würbel H. (2014). Refinement of experimental design and conduct in laboratory animal research. ILAR J. 55 (3), 383–391. 10.1093/ilar/ilu037 25541540

[B5] Baldessarini R. J. Tondo L. (2019). Effects of treatment discontinuation in clinical psychopharmacology. Psychother. Psychosom. 88 (2), 65–70. 10.1159/000497334 30923289

[B6] Bedard P. L. Hyman D. M. Davids M. S. Siu L. L. (2020). Small molecules, big impact: 20 years of targeted therapy in oncology. Lancet 395 (10229), 1078–1088. 10.1016/S0140-6736(20)30164-1 32222192

[B7] Beghi E. Giussani G. Grosso S. Iudice A. La Neve A. Pisani F. (2013). Withdrawal of antiepileptic drugs: guidelines of the Italian league against epilepsy. Epilepsia 54 (Suppl. 7), 2–12. 10.1111/epi.12305 24099051

[B8] Bozec A. Formento P. Fischel J. L. Etienne-Grimaldi M. C. Milano G. (2010). Tapered dose *versus* constant drug exposure to anti-EGFR drugs on head-and-neck cancer xenografts. A comparison between cetuximab and gefitinib. Oral Oncol. 46 (3), 172–177. 10.1016/j.oraloncology.2009.11.010 20156700

[B9] Cai D. Gui D. Huang Y. Zhou W. (2024). Respiratory failure induced by multi-drug combination in buprenorphine dependence: a case report. Chin. J. Drug Dependence 33 (02), 174–177. 10.13936/j.cnki.cjdd1992.2024.02.016

[B10] Cardona V. Ansotegui I. J. Ebisawa M. El-Gamal Y. Fernandez Rivas M. Fineman S. (2020). World allergy organization anaphylaxis guidance 2020. World Allergy Organ J. 13 (10), 100472. 10.1016/j.waojou.2020.100472 33204386 PMC7607509

[B11] Chen J. Shen T. Li J. Ling S. Yang Z. Wang G. (2021). The Chinese clinical practice guideline on liver transplantation for hepatocellular carcinoma. Pract. J. Organ Transplantation (Electronic Version) 10 (06), 481–489+480.

[B12] Chen Y. Huang Y. Shi W. (2010). Effect of Shenkang-Ⅱ on hypothalamo-pituitary-adrenal axis in nephrotic syndrome rats during glucocorticoids withdrawal. Chin. J. Inf. Traditional Chin. Med. (2). 10.3969/j.issn.1005-5304.2010.02.011

[B13] China Association Against Epilepsy (2015). Clinical guidelines: epilepsy fascicle. Beijing, China: People’s Medical Publishing House.

[B14] Chinese Education Association of Chronic Airway Diseases; China Asthma AllianceChina Asthma Alliance (2024). Chinese expert consensus on the diagnosis and management of severe asthma (2024 edition). Zhonghua Yi Xue Za Zhi 104 (20), 1759–1789. 10.3760/cma.j.cn112137-20231117-01120 38782746

[B15] Chinese Society of Organ Transplantation (2019). Technical specifications for the clinical application of immunosuppressive agents in organ transplantation (2019). Organ Transplant. 10 (3), 213–226. 10.3969/j.issn.1674-7445.2019.03.001

[B16] Consensus Expert Group on the Emergency Use of Glucocorticoids (2020). Expert consensus on the emergency use of glucocorticoids. Chin. J. Emerg. Med. 29 (6), 765–772. 10.3760/cma.j.issn.1671-0282.2020.06.005

[B17] Dagar A. Falcone T. (2020). Psychiatric comorbidities in pediatric epilepsy. Curr. Psychiatry Rep. 22 (12), 77. 10.1007/s11920-020-01195-8 33128638

[B18] Das Thakur M. Salangsang F. Landman A. S. Sellers W. R. Pryer N. K. Levesque M. P. (2013). Modelling vemurafenib resistance in melanoma reveals a strategy to forestall drug resistance. Nature 494 (7436), 251–255. 10.1038/nature11814 23302800 PMC3930354

[B19] Dirnagl U. Endres M. (2014). Found in translation: preclinical stroke research predicts human pathophysiology, clinical phenotypes, and therapeutic outcomes. Stroke 45 (5), 1510–1518. 10.1161/STROKEAHA.113.004075 24652307

[B20] Duy P. Q. Krauss G. L. Crone N. E. Ma M. Johnson E. L. (2020). Antiepileptic drug withdrawal and seizure severity in the epilepsy monitoring unit. Epilepsy Behav. 109, 107128. 10.1016/j.yebeh.2020.107128 32417383

[B21] Fanouriakis A. Kostopoulou M. Andersen J. Aringer M. Arnaud L. Bae S. C. (2024). EULAR recommendations for the management of systemic lupus erythematosus: 2023 update. Ann. Rheum. Dis. 83 (1), 15–29. 10.1136/ard-2023-224762 37827694

[B22] Feng T. (2013). The influencing factors of the recurrence of behavioral and psychological in patients with alzheimer's disease. J. Brain Nerv. Dis. 21 (02), 110–112. 10.3969/j.issn.1006-351X.2013.02.011

[B23] Gao J. Liu G. (2023). Drug therapies for autoimmune diseases Chin. J. Clin. Ed. 17 (12), 1209–1211. 10.3877/cma.j.issn.1674-0785.2023.12.001

[B24] Grace P. M. Galer E. L. Strand K. A. Corrigan K. Berkelhammer D. Maier S. F. (2019). Repeated morphine prolongs postoperative pain in Male rats. Anesth. Analg. 128 (1), 161–167. 10.1213/ANE.0000000000003345 29596097 PMC7054903

[B25] Gu X. Lu Y. Di Q. (2023). Research progress of drug withdrawal in patients after epilepsy surgery. Chin. J. Nerv. Ment. Dis. 49 (12), 753–757. 10.3969/j.issn.1002-0152.2023.12.009

[B26] He F. (2006). Experimental study on the effect of jiajiawu meiwan on airway inflammation and HPA axis in asthmatic rats after hormonal intervention [D]. Beijing, China: Beijing University of Chinese Medicine.

[B27] Hodsman A. B. Watson P. H. Drost D. Holdsworth D. Thornton M. Hock J. (1999). Assessment of maintenance therapy with reduced doses of PTH(1-34) in combination with a raloxifene analogue (LY117018) following anabolic therapy in the ovariectomized rat. Bone 24 (5), 451–455. 10.1016/s8756-3282(99)00015-0 10321904

[B28] Horowitz M. A. Jauhar S. Natesan S. Murray R. M. Taylor D. (2021). A method for tapering antipsychotic treatment that may minimize the risk of relapse. Schizophr. Bull. 47 (4), 1116–1129. 10.1093/schbul/sbab017 33754644 PMC8266572

[B29] Horowitz M. A. Taylor D. (2022). How to reduce and stop psychiatric medication. Eur. Neuropsychopharmacol. 55, 4–7. 10.1016/j.euroneuro.2021.10.001 34688998

[B30] Jingyou Li Li J. (2023). Analysis of influencing factors of relapse after drug withdrawal in children with epilepsy. Smart Healthc. 9 (29), 73–76+80. 10.19335/j.cnki.2096-1219.2023.29.018

[B31] Julian I. Iwamoto T. (2021). Investigation of biomarkers and handling strategy of erlotinib-induced skin rash in rats. Biol. Pharm. Bull. 44 (8), 1050–1059. 10.1248/bpb.b21-00112 34334490

[B32] Kalemkerian G. P. Narula N. Kennedy E. B. Biermann W. A. Donington J. Leighl N. B. (2018). Molecular testing guideline for the selection of patients with lung cancer for treatment with targeted tyrosine kinase inhibitors: American society of clinical oncology endorsement of the college of American pathologists/international association for the study of lung cancer/association for molecular pathology clinical practice guideline update. J. Clin. Oncol. 36 (9), 911–919. 10.1200/JCO.2017.76.7293 29401004

[B33] Karalok Z. S. Guven A. Öztürk Z. Gurkas E. (2020). Risk factors for recurrence after drug withdrawal in childhood epilepsy. Brain Dev. 42 (1), 35–40. 10.1016/j.braindev.2019.08.012 31521420

[B34] Kaur R. Afzal M. Kazmi I. Ahamd I. Ahmed Z. Ali B. (2013). Polypharmacy (herbal and synthetic drug combination): a novel approach in the treatment of type-2 diabetes and its complications in rats. J. Nat. Med. 67 (3), 662–671. 10.1007/s11418-012-0720-5 23151907

[B35] Kidney Disease: Improving Global Outcomes (KDIGO) Glomerular Diseases Work Group (2021). KDIGO 2021 clinical practice guideline for the management of glomerular diseases. Kidney Int. 100 (4S), S1–S276. 10.1016/j.kint.2021.05.021 34556256

[B36] Kilkenny C. Browne W. J. Cuthill I. C. Emerson M. Altman D. G. (2010). Improving bioscience research reporting: the ARRIVE guidelines for reporting animal research. PLoS Biol. 8 (6), e1000412. 10.1371/journal.pbio.1000412 20613859 PMC2893951

[B37] Kong X. Kuilman T. Shahrabi A. Boshuizen J. Kemper K. Song J. Y. (2017). Cancer drug addiction is relayed by an ERK2-dependent phenotype switch. Nature 550 (7675), 270–274. 10.1038/nature24037 28976960 PMC5640985

[B38] Ladd A. D. Duarte S. Sahin I. Zarrinpar A. (2024). Mechanisms of drug resistance in HCC. Hepatology 79 (4), 926–940. 10.1097/HEP.0000000000000237 36680397

[B39] Lamberink H. J. Otte W. M. Geleijns K. Braun K. P. J. (2015). Antiepileptic drug withdrawal in medically and surgically treated patients: a meta-analysis of seizure recurrence and systematic review of its predictors. Epileptic Disord. 17 (3), 211–228. 10.1684/epd.2015.0764 26292909

[B40] Li L. Ma X. (2017). Interpretation of the Chinese guidelines for the prevention and treatment of depressive disorders. Chin. J. Psychiatry 50 (003), 21. 10.3760/cma.j.issn.1006-7884.2017.03.002

[B80] Li N. (2018). Intervention Effects of Different Administration Routes of Simvastatin on Inflammatory Factors in Peripheral Blood of Fracture Rats [D]. Shanxi, China: Shanxi Medical University.

[B41] Li N. Wu F. Wang F. Luo T. Zhang L. Xu L. (2019). Effect of simvastatin on inflammatory cytokine levels of peripheral blood in a rat fracture model by different administration methods. Beijing J. Stomatology 27 (01), 20–23.

[B79] Li Z. Li L. Ran J. Zhang S. Liu J. Liu D. (2010). Dynamically observed histopathologic changes of acute rejection in rat orthotopic liver transplantation model after tacrolimus discontinued. Chin. J. Bases Clin. General Surg. 12.

[B43] Liu C. Hsieh M. Chien Y. Liu C. Lin Y. Hwang T. (2023). Dose-tapering trajectories in patients with remitted psychosis undergoing guided antipsychotic reduction to reach minimum effective dose. Eur. Psychiatry. 66 (1), e66. 10.1192/j.eurpsy.2023.2440 37578111 PMC10594210

[B44] Liu R. Yuan Y. Zhang W. Wang P. (2012). Impacts of herba epimedii and fructus ligustri lucidi in asthma rat models with hormone intervention at the withdrawal stages. World J. Integr. Traditional West. Med. 7 (7). 10.13935/j.cnki.sjzx.2012.07.028

[B45] Long D. R. Lihn A. L. Friedrich S. Scheffenbichler F. T. Safavi K. C. Burns S. M. (2018). Association between intraoperative opioid administration and 30-day readmission: a pre-specified analysis of registry data from a healthcare network in new England. Br. J. Anaesth. 120 (5), 1090–1102. 10.1016/j.bja.2017.12.044 29661386

[B46] Ma X. Liu D. Liu X. Zhang B. (2024). Effect of remifentanil sequential withdrawal on pain and quality of awakening after cervical spine surgery. J. Cervicodynia And Lumbodynia 45 (03), 578–581. 10.3969/j.issn.1005-7234.2024.03.044

[B47] Macenski M. J. Schaal D. W. Cleary J. Thompson T. (1994). Changes in food-maintained progressive-ratio responding of rats following chronic buprenorphine or methadone administration. Pharmacol. Biochem. Behav. 47 (2), 379–383. 10.1016/0091-3057(94)90027-2 8146232

[B48] Meng H. Ji Z. Guo D. (2023). Perioperative withdrawal malignant syndrome in parkinson's disease: a case report. Chin. J. Pharmacoepidemiol. 32 (08), 941–944. 10.19960/j.issn.1005-0698.202308013

[B49] Mo L. Zheng P. (2024). Expert consensus on clinical pharmacy of immune Drugs[J/OL]. Pharm. Today 1-20. Available online at: http://kns.cnki.net/kcms/detail/44.1650.R.20231010.0917.002.html.

[B50] National Research Council of the National Academies (2011). Guide for the care and use of laboratory animal. Eighth Edition. USA: The National Academies Press.

[B51] Ogden B. D. (1996). Principles of animal research: replacement, reduction, refinement, and responsibility. Anim. Law Rev. 2, 167–170. Available online at: https://lawcommons.lclark.edu/alr/vol2/iss1/10

[B52] Ottaviano M. Giunta E. F. Tortora M. Curvietto M. Attademo L. Bosso D. (2021). BRAF gene and melanoma: back to the future. Int. J. Mol. Sci. 22 (7), 3474. 10.3390/ijms22073474 33801689 PMC8037827

[B53] Peng L. Nie Y. Zhou J. Wu L. Chen X. Wang F. (2024). Withdrawal of immunosuppressants and low-dose steroids in patients with stable IgG4-RD (WInS IgG4-RD): an investigator-initiated, multicentre, open-label, randomised controlled trial. Ann. Rheum. Dis. 83 (5), 651–660. 10.1136/ard-2023-224487 38216319

[B54] Peters M. D. J. Marnie C. Tricco A. C. Pollock D. Munn Z. Alexander L. (2021). Updated methodological guidance for the conduct of scoping reviews. JBI Evid. Implement 19 (1), 3–10. 10.1097/XEB.0000000000000277 33570328

[B55] Pound P. Ritskes-Hoitinga M. (2018). Is it possible to overcome issues of external validity in preclinical animal research? Why Most animal models are bound to fail. J. Transl. Med. 16 (1), 304. 10.1186/s12967-018-1678-1 30404629 PMC6223056

[B56] Qian K. (2024). Research progress in surgical pain management under the ERAS philosophy. Mod. Nurse 31 (01), 4–7. 10.19793/j.cnki.1006-6411.2024.03.002

[B57] Reidenberg M. M. (2011). Drug discontinuation effects are part of the pharmacology of a drug. J. Pharmacol. Exp. Ther. 339 (2), 324–328. 10.1124/jpet.111.183285 21849624 PMC3200000

[B58] Russell W. M. S. Burch R. (1959). The principles of humane experimental technique. London: Methuen.

[B59] Sbiera S. Dexneit T. Reichardt S. D. Michel K. D. van den Brandt J. Schmull S. (2011). Influence of short-term glucocorticoid therapy on regulatory T cells *in vivo* . PLoS One 6 (9), e24345. 10.1371/journal.pone.0024345 21912688 PMC3166315

[B60] Sereno J. Vala H. Nunes S. Rocha-Pereira P. Carvalho E. Alves R. (2015). Cyclosporine A-induced nephrotoxicity is ameliorated by dose reduction and conversion to sirolimus in the rat. J. Physiol. Pharmacol. 66 (2), 285–299. 25903959

[B61] Shi B. Yuan M. (2016). Guidelines on immunosuppressive therapy for renal transplant recipients in China (2016). Organ Transplant. 7 (05), 327–331. 10.3969/j.issn.1674-7445.2016.05.001

[B62] Smolen J. S. Landewé R. B. M. Bergstra S. A. Kerschbaumer A. Sepriano A. Aletaha D. (2023). EULAR recommendations for the management of rheumatoid arthritis with synthetic and biological disease-modifying antirheumatic drugs: 2022 update. Ann. Rheum. Dis. 82 (1), 3–18. 10.1136/ard-2022-223356 36357155

[B63] Strozzi I. Nolan S. J. Sperling M. R. Wingerchuk D. M. Sirven J. (2015). Early versus late antiepileptic drug withdrawal for people with epilepsy in remission. Cochrane Database Syst. Rev. 2015 (2), CD001902. 10.1002/14651858.CD001902.pub2 25922863 PMC7061653

[B64] Su K. Jiang L. Guo Y. (2014). Effect of wuzi sanhuang formula on the function of hypothalamic-pituitary-adrenal axis in mice with hormone-dependent asthma. J. Traditional Chin. Med. 55 (2), 153–156. 10.13288/j.11-2166/r.2014.02.017

[B65] Taylor A. L. Watson C. J. Bradley J. A. (2005). Immunosuppressive agents in solid organ transplantation: mechanisms of action and therapeutic efficacy. Crit. Rev. Oncol. Hematol. 56 (1), 23–46. 10.1016/j.critrevonc.2005.03.012 16039869

[B66] Totzeck M. Mincu R. I. Rassaf T. (2017). Cardiovascular adverse events in patients with cancer treated with bevacizumab: a meta-analysis of more than 20 000 patients. J. Am. Heart Assoc. 6 (8), e006278. 10.1161/JAHA.117.006278 28862931 PMC5586462

[B67] Tricco A. C. Lillie E. Zarin W. O'Brien K. K. Colquhoun H. Levac D. (2018). PRISMA extension for scoping reviews (PRISMA-ScR): checklist and explanation. Ann. Intern Med. 169 (7), 467–473. 10.7326/M18-0850 30178033

[B68] Vorderwülbecke B. J. Kirschbaum A. Merkle H. Senf P. Holtkamp M. (2019). Discontinuing antiepileptic drugs in long-standing idiopathic generalised epilepsy. J. Neurol. 266 (10), 2554–2559. 10.1007/s00415-019-09457-z 31267208

[B69] Wang X. (2010). Study of the effect of adrenal glucocorticoids on their receptors, heat shock protein 90 and histone deacetylase 6 and intervention with herbal medicine [D]. Liaoning, China: China Medical University.

[B70] Xiong W. Wei Q. Liu X. (2005). Introduction to systematic reviews of animal studies. Chin. J. Evidence-Based Med. (02), 161–163+173. 10.3969/j.issn.1672-2531.2005.02.016

[B71] Yan T. (2016). Effect of three-step sequential method on TGF-β_1/Smad signaling pathway in patients with hormone-dependent asthma and in rats with airway remodeling model during hormone withdrawal [D]. Beijing, China: Beijing University Of Chinese Medicine.

[B72] Yang X. He J. Guo X. Tian R. (2023). Amino acid metabolism characteristics of banxia baizhu tianma decoction in realizing drug withdrawal based on transcriptomic analysis. China J. Chin. Materia Medica 48 (9), 2512–2521. 10.19540/j.cnki.cjcmm.20230117.702 37282880

[B42] Yao L. Ruan J. Lin Z. Yuan Y. Qiu S. Jiang Z. (2022). The combined inhibitory effect of intermittent therapy plus ERK1/2-MITF pathway activation on the proliferation of drug-resistant melanoma. China Trop. Med. 22 (02), 106–111+122. 10.13604/j.cnki.46-1064/r.2022.02.03

[B73] Zhang T. (2015). Alzheimer's disease: stopping medication at will is not advisable [N]. China Pharmaceutical News. [Beijing, China] 18 Sept., p. 1.

[B74] Zhang X. Cai N. Zhang W. Dai C. (2022). Effect of remifentanil withdrawal gradually on pain and complications after ACDF. J. Cervicodynia And Lumbodynia 43 (02), 222–224. 10.3969/j.issn.1005-7234.2022.02.019

[B75] Zhao J. Shi S. (2015). Guidelines for the prevention and treatment of schizophrenia in China [M]. Beijing, China: Chinese Medical Multimedia Press.

[B76] Zhou D. Jin Y. Xu T. Hou Z. (2019). Study on the promoting effect of simvastatin on the tibial fracture healing of rat and its related signaling pathway. Chin. J. Mod. Appl. Pharm. 36 (18). 10.13748/j.cnki.issn1007-7693.2019.18.006

[B77] Zhou X. Lin G. (2022). Advances in research on drug withdrawal in childhood epilepsy. J. Shantou Univ. Med. Coll. 35 (01), 62–64. 10.13401/j.cnki.jsumc.2022.01.018

[B78] Zhu C. Chen Y. Liu M. Chen Y. (2021). Efficacy and safety ofapatinib monotherapy in patients with advanced malignancie. J. Mod. Oncol. 29 (20), 3649–3653. 10.3969/j.issn.1672-4992.2021.20.030

