# Supplementary material for: Drug tapering in animal research: current practices and challenges
Source: Front Pharmacol. 2025 Aug 13;16:1544784. doi: 10.3389/fphar.2025.1544784 (PMC12380909; doi:10.3389/fphar.2025.1544784)
Supplement: Supplementary file 1 [file Table1.docx]

Supplementary Material

**Search queries**

The search queries for PubMed, Web of Science, China National Knowledge Infrastructure Database (CNKI), Wanfang Database, VIP Database for Chinese Technical Periodicals (VIP), and the Chinese Biomedical Literature Database (SinoMed) are presented in Tables 1 through 6.

**Table 1** Search queries for PubMed

| Number | Search terms |
| --- | --- |
| 1 | “Drug Tapering” [MeSH Terms] |
| 2 | “Dose Tapering” |
| 3 | “Tapering, Dose” |
| 4 | “Dose Reduction” |
| 5 | “Dose Reductions” |
| 6 | “Reduction, Dose” |
| 7 | “Reductions, Dose” |
| 8 | “medicine reduction” |
| 9 | OR/1-8 |
| 10 | "Rats"[MeSH Terms] |
| 11 | "Rat" |
| 12 | "Rattus" |
| 13 | "Rats, Laboratory" |
| 14 | "Laboratory Rat" |
| 15 | "Laboratory Rats" |
| 16 | "Rat, Laboratory" |
| 17 | "Rattus norvegicus" |
| 18 | "norvegicus, Rattus" |
| 19 | "Rats, Norway" |
| 20 | "Norway Rat" |
| 21 | "Norway Rats" |
| 22 | "Rat, Norway" |
| 23 | OR/10-22 |
| 24 | "mice"[MeSH Terms] |
| 25 | "Mus" |
| 26 | "Mouse" |
| 27 | "Mice, Laboratory" |
| 28 | "Laboratory Mice" |
| 29 | "Mouse, Laboratory" |
| 30 | "Laboratory Mouse" |
| 31 | "Mouse, Swiss" |
| 32 | "Swiss Mouse" |
| 33 | "Swiss Mice" |
| 34 | "Mice, Swiss" |
| 35 | "Mus domesticus" |
| 36 | "Mus musculus domesticus" |
| 37 | "domesticus, Mus musculus" |
| 38 | "Mus musculus" |
| 39 | "Mouse, House" |
| 40 | "House Mouse" |
| 41 | "Mice, House" |
| 42 | "House Mice" |
| 43 | OR/24-42 |
| 44 | "Animal Experimentation"[MeSH Terms]) |
| 45 | "Experimentation, Animal" |
| 46 | "Animal Experimental Use" |
| 47 | "Animal Research" |
| 48 | "Research, Animal") |
| 49 | "Animal Experiments" |
| 50 | "Animal Experiment" |
| 51 | "Experiment, Animal" |
| 52 | "Experiments, Animal" |
| 53 | OR/44-52 |
| 54 | #23 OR #43 OR #53 |
| 55 | #9 AND #54 604 results |

**Table 2** Search queries for Web of Science

| Number | Search terms |
| --- | --- |
| 1 | TS=((((((("Animal Experimentation") OR ("Animal Experimental Use")) OR ("Animal Research")) OR ("Animal Experiments")) OR ("Animal Experiment")) OR ("Experiments, Animal"))) |
| 2 | TS=(((((((((“Rats”) OR (“Rat”)) OR (“Rattus”)) OR ("Laboratory Rat")) OR ("Laboratory Rats")) OR ("Rattus norvegicus")) OR ("Norway Rat")) OR ("Norway Rats"))) |
| 3 | TS=((((((((((((("mice") OR ("Mus")) OR ("Mouse")) OR ("Laboratory Mice")) OR ("Laboratory Mouse")) OR ("Swiss Mouse")) OR ("Swiss Mice")) OR ("Mus domesticus")) OR ("Mus musculus domesticus")) OR ("Mus musculus")) OR ("House Mouse")) OR ("House Mice"))) |
| 4 | TS=(((((("Drug Tapering") OR ("Dose Tapering")) OR ("Dose Reduction")) OR ("Dose Reductions")) OR ("medicine reduction") )) |
| 5 | #1 OR #2 OR #3 |
| 6 | #4 AND #5 486 results |

**Table 3** Search queries for CNKI

| Number | Search terms |
| --- | --- |
| 1 | 篇关摘=减药 + 撤药 + 减撤 + 撤减 + 减停 + 撤停 |
| 2 | 篇关摘=动物 + 鼠 + 鱼 + 犬 + 狗 + 兔 + 猴 + 猪 + 鸡 + 羊 + 马 + 鸽 |
| 3 | #1 AND #2 381 results |

**Table 4** Search queries for Wanfang

| Number | Search terms |
| --- | --- |
| 1 | 主题="减药" or "撤药" or "减撤" or "减停" or "撤减" or "撤停" |
| 2 | 主题=动物 or 鼠 or 鱼 or 犬 or 狗 or 兔 or 猴 or 猪 or 鸡 or 羊 or 马 or 鸽 |
| 3 | #1 AND #2 489 results |

**Table 5** Search queries for VIP

| Number | Search terms |
| --- | --- |
| 1 | 摘要="减药" or "撤药" or "减撤" or "减停" or "撤减" or "撤停" |
| 2 | 摘要=动物 or 鼠 or 鱼 or 犬 or 狗 or 兔 or 猴 or 猪 or 鸡 or 羊 or 马 or 鸽 |
| 3 | #1 AND #2 662 results |

**Table 6** Search queries for SinoMed

| Number | Search terms |
| --- | --- |
| 1 | 常用字段="减药" or "撤药" or "减撤" or "减停" or "撤减" or "撤停" |
| 2 | 常用字段=动物 or 鼠 or 鱼 or 犬 or 狗 or 兔 or 猴 or 猪 or 鸡 or 羊 or 马 or 鸽 |
| 3 | #1 AND #2 175 results |
